# Formulating formation mechanism of natural gas hydrates

**DOI:** 10.1038/s41598-017-06717-8

**Published:** 2017-07-25

**Authors:** Avinash V. Palodkar, Amiya K. Jana

**Affiliations:** Energy and Process Engineering Laboratory, Department of Chemical Engineering, Indian Institute of Technology, Kharagpur, 721302 India

## Abstract

A large amount of energy, perhaps twice the total amount of all other hydrocarbon reserves combined, is trapped within gas hydrate deposits. Despite emerging as a potential energy source for the world over the next several hundred years and one of the key factors in causing future climate change, gas hydrate is poorly known in terms of its formation mechanism. To address this issue, a mathematical formulation is proposed in the form of a model to represent the physical insight into the process of hydrate growth that occurs on the surface and in the irregular nanometer-sized pores of the distributed porous particles. To evaluate the versatility of this rigorous model, the experimental data is used for methane (CH_4_) and carbon dioxide (CO_2_) hydrates grown in different porous media with a wide range of considerations.

## Introduction

The world is addicted to hydrocarbons that are naturally available in the form of oil, coal and gas, which meet about 80% of our current energy needs^1^. Burning these fossil fuels has devastating side effects through the emission of carbon dioxide (CO_2_) to the atmosphere. However, it is projected that about 78% of the global energy requirement at 2040 will come from these carbon based sources^2^. Fortunately, there is a huge reserve of natural methane (CH_4_) gas locked away under deep seabed and vast swathes of permafrost^3^. To meet the consistently growing energy demand and control the increasing concentration of carbon dioxide in the atmosphere, gas hydrate can play a crucial role. In this light, an ambitious CH_4_ – CO_2_ swapping process is proposed in literature^4^ that aims to dislodge methane from the hydrate structure cavities by carbon dioxide gas. At certain pressure and temperature conditions, the CO_2_ hydrate provides more stable form than the CH_4_ hydrate^4^.

In the CH_4_-CO_2_ replacement process, carbon dioxide is directly injected into methane hydrate layers^5^. With this, firstly, the hydrate – gas equilibrium gets disturbed due to the change in vapor composition. In the subsequent step, decomposition of hydrate occurs along with the reformation of a transient mixed hydrate at the surface of the original hydrate particle. Finally, a new equilibrium is established. This guest gas basically forms a thermodynamically preferred gas hydrate and replaces the methane molecule within the hydrate cavity^6^. During the solid-liquid-solid transition, there is no apparent dissociation noticed, which indicates that the geomechanical stability remains unaffected^7^. As indicated, in addition to CH_4_ production, this swapping phenomenon is used for stable long term CO_2_ storage^8^. This, in turn, leads to maintain structural integrity and reduce seepage since CO_2_ hydrate itself acts as an additional sealing layer^6^. At this point, it is interesting to note that the P-wave velocity (i.e., compressional wave velocity) is measured to have the information of the stiffness evolution of hydrate-bearing sediments that is related to the reservoir stability^9^.

Gas hydrates are ice-like crystalline three-dimensional (3D) structure with gas molecules trapped inside the hydrogen bonded microscopic water cages. They typically form when small gas molecules (<0.9 nm)^10^ come to contact with water at high pressure and low temperature. From scientific and industrial perspective, gas hydrates are relevant in climate change modeling^11^, storage of natural gas and hydrogen^12, 13^, CO_2_ sequestration^14^, gas separation^15^, and seawater desalination^16^, among others. On the other side, their decomposition and subsequent release of methane gas may cause submarine geohazards, such as sediment instabilities and slope failures, leading to debris flows, slumps, slides, and possible tsunamis^17^. Again, escaping CH_4_ from dissociated gas hydrate into the atmosphere has more severe greenhouse effect since it has the ability to absorb infrared radiation approximately 25 times more efficiently than CO_2_ (ref. 6).

Depending on the type and size of guest molecules, there are two common hydrate structures formed in the name of Structure I (sI) and structure II (sII)^18^. Among them, sI crystal contains two small 5^12^ cavities (average cavity radius = 3.95 Å) and six large 5^12^6^2^ cavities (average cavity radius = 4.33 Å) per unit cell, whereas sII crystal has sixteen small 5^12^ cavities (average cavity radius = 3.91 Å) and eight large 5^12^6^4^ cavities (average cavity radius = 4.73 Å) per unit cell^10^. It is worth noticing that cubic structure I occurs with small (0.4–0.55 nm) guests and it predominates in the Earth’s natural environments; and cubic structure II usually forms with larger (0.6–0.7 nm) guests in the man-made environments^10^.

Despite worldwide abundance of gas hydrate and its potential as an energy source of the future, the growth kinetics of this crystalline structure remains poorly understood^19^. To address this issue, in this study, a mathematical formulation is developed in the form of a physical model that is capable and versatile enough in precisely explaining the formation kinetics of various gas hydrates. Perhaps, there is no such generalized formulation reported in literature to predict the real-time growth behavior.

## Results

### Formulating hydrate formation kinetics

Hydrate formation seems to occur at the interface between the bulk guest and aqueous phases. This formation may be driven by the difference existed in temperature^20, 21^, pressure^22^, gas composition^23^ or in fugacity^24–28^. Here, the driving force is proposed as the difference in chemical potential of water in the aqueous phase (${{\rm{\Delta }}\mu }_{{\rm{w}}}^{{\rm{A}}}$) and water in the hydrate phase (${{\rm{\Delta }}\mu }_{{\rm{w}}}^{{\rm{H}}}$).

Clathrate hydrates mostly form in the interstitial pore space between porous particles^29^. Accordingly, the consumption of guest gas is proposed to vary proportionally with the said chemical potential difference, along with the water transformation rate and the total particle surface area (*A*). This yields,

1 $$\frac{{\rm{d}}{n}_{{\rm{gg}}}}{{\rm{d}}t}={K}_{{\rm{0}}}\exp (\frac{-{\rm{\Delta }}{E}_{{\rm{a}}}}{RT})A(\frac{{\rm{\Delta }}{\mu }_{{\rm{w}}}^{{\rm{A}}}}{RT}-\frac{{\rm{\Delta }}{\mu }_{{\rm{w}}}^{{\rm{H}}}}{RT}){n}_{{{\rm{H}}}_{{\rm{2}}}{\rm{O}},{\rm{A}}}$$

in which, *n*_gg_ is the mole of guest gas consumed during hydrate formation, ${n}_{{{\rm{H}}}_{{\rm{2}}}{\rm{O}},{\rm{A}}}$ the residual mole of water, *K*_*0*_ the intrinsic rate constant, Δ*E*_a_ the activation energy, *T* the temperature and *R* the universal gas constant (8.314 J.mol^−1^.K^−1^). This modeling equation is formulated by assuming the first-order reaction kinetics for water transformation in terms of ${n}_{{{\rm{H}}}_{{\rm{2}}}{\rm{O}},{\rm{A}}}$. Further, it considers that the kinetic constant (*K*) is temperature dependent and represented by an Arrhenius-type equation as:

2 $$K={K}_{{\rm{0}}}\exp (\frac{-{\rm{\Delta }}{E}_{{\rm{a}}}}{RT})$$

As time progresses, the hydrate film increases in size and it acts as a barrier at the interface. This leads to decrease the contact area involved in hydrate growth between the bulk guest and aqueous phase. Accordingly, the concept of effective surface area (*A*_*e*_) is introduced^29^ as:

3 $${A}_{e}=\beta A$$

The surface area adjustment factor, *β* lies between 0 and 1. Actually, *β* needs to be tuned by the use of an optimization technique.

Now, one needs to replace ${n}_{{{\rm{H}}}_{{\rm{2}}}{\rm{O}},{\rm{A}}}$ by the mole of guest gas (*n*_gg_), for which, the following equation can be used:

4 $${n}_{{{\rm{H}}}_{{\rm{2}}}{\rm{O}},{\rm{A}}}={n}_{{{\rm{H}}}_{{\rm{2}}}{\rm{O}},{\rm{T}}}-{n}_{{\rm{H}}}{n}_{{\rm{gg}}}$$

This is obtained by using the following correlations:

5 $${n}_{{{\rm{H}}}_{{\rm{2}}}{\rm{O}},{\rm{T}}}={n}_{{{\rm{H}}}_{{\rm{2}}}{\rm{O}},{\rm{A}}}+{n}_{{{\rm{H}}}_{{\rm{2}}}{\rm{O}},{\rm{H}}}$$

6 $${n}_{{{\rm{H}}}_{{\rm{2}}}{\rm{O}},{\rm{H}}}={n}_{{\rm{H}}}{n}_{{\rm{gg}}}$$

where, ${n}_{{{\rm{H}}}_{{\rm{2}}}{\rm{O}},{\rm{T}}}$ refers to the total number of moles of water initially present, *n*_*H*_ the hydration number and ${n}_{{{\rm{H}}}_{{\rm{2}}}{\rm{O}},{\rm{H}}}$ the number of moles of water converted to hydrate.

Integrating Equation (1) and rearranging, one obtains^29^

7 $$\frac{{n}_{{\rm{gg}}}}{{n}_{{{\rm{H}}}_{{\rm{2}}}{\rm{O}},{\rm{T}}}}=\frac{\alpha }{{n}_{{\rm{H}}}}\{{\rm{1}}-\exp [-{n}_{{\rm{H}}}{K}_{{\rm{0}}}\exp (\frac{-{\rm{\Delta }}{E}_{{\rm{a}}}}{RT})\frac{\beta }{RT}A({\mu }_{{\rm{w}}}^{{\rm{H}}}-{\mu }_{{\rm{w}}}^{{\rm{A}}})t]\}$$

Here, *t* denotes the time and *α* an adjustable parameter that is defined as the ratio of highest value of the net amount of guest gas consumed during the hydrate formation and the total amount of that gas ideally occupied in all cavities.

Targeting to the formation of CH_4_ and CO_2_ gas hydrates, both of which are sI type hydrate, one can compute ${\mu }_{w}^{{\rm{H}}}$ from^30^:

8 $$\frac{\Delta {\mu }_{{\rm{w}}}^{{\rm{H}}}}{RT}=-[\sum _{i=1}^{2}{v}_{i}\,\mathrm{ln}(1-\sum _{j=1}^{{N}_{{\rm{c}}}}{\theta }_{ij})]$$

Here, *v*_*i*_ stands for the number of cavities or cages of type *i* per water molecule in the hydrate phase and *θ*_*ij*_ the fractional occupancy of *i* type cavity with *j* type guest molecule.

Again, ${\mu }_{w}^{{\rm{A}}}$ is formulated with^30^:

9 $$\frac{{\rm{\Delta }}{\mu }_{{\rm{w}}}^{{\rm{A}}}(T,P)}{RT}=\frac{{\rm{\Delta }}{\mu }_{{\rm{w}}}^{{\rm{0}}}(T,0)}{R{T}_{{\rm{0}}}}-{\int }_{{T}_{0}}^{T}\frac{{\rm{\Delta }}{h}_{{\rm{w}}}^{{\rm{A}}}(T)}{R{T}^{2}}{\rm{d}}T+{\int }_{0}^{P}\frac{{\rm{\Delta }}{V}_{{\rm{w}}}^{{\rm{A}}}}{R{T}^{2}}{\rm{d}}P-\,\mathrm{ln}({a}_{w})$$

in which, *T*_*0*_ is the reference temperature (=273.15 K), ${\rm{\Delta }}{\mu }_{{\rm{w}}}^{{\rm{0}}}(T,0)$ the standard chemical potential difference of water for gas hydrate at reference temperature and absolute zero pressure, and it is adopted as 1202 J.mol^−1^ (ref. 31), $\Delta {V}_{w}^{{\rm{A}}}$ the difference between molar volume of water in hydrate and aqueous phase, $\Delta {h}_{w}^{{\rm{A}}}$ the enthalpy difference between empty hydrate lattice and liquid water, and *a*_*w*_ the activity of water.

**Figure 1:**
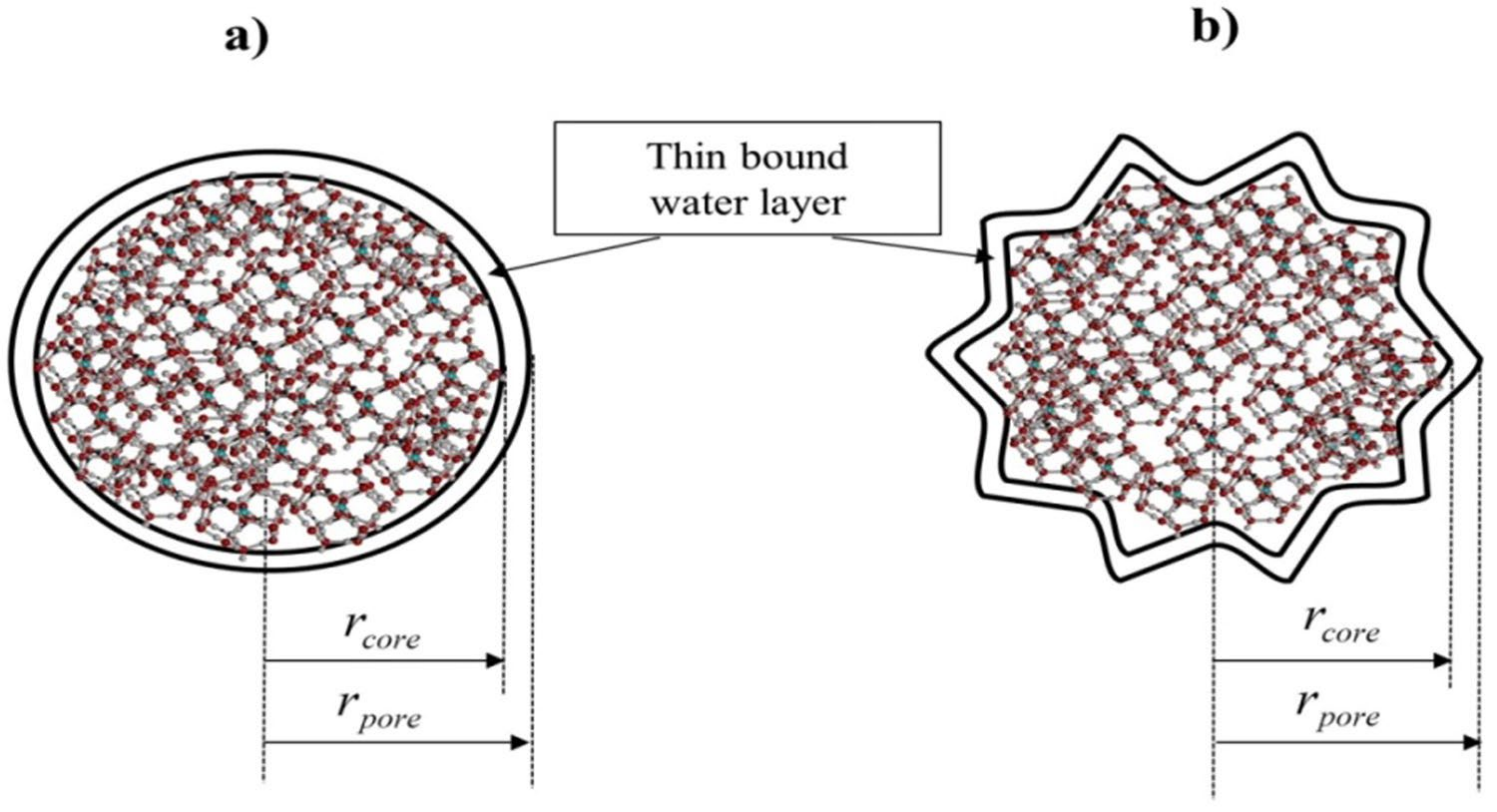
Gas hydrate structure in (**a**) regular and (**b**) irregular pore of a porous medium.

Along with the interstitial pore space between porous materials, gas hydrates are likely to form and grow inside the nanometer-sized pores of those materials. In this regard, one can see the experimental evidence provided in ref. 32. To formulate this growth kinetics, it is quite realistic to consider that the pores are irregular in shape and size (schematic in Fig. 1), and they are present in the distributed particles. Further, it is considered that a thin bound water monolayer having a thickness of 0.4 nm^33^ is present on the pore wall. With these, assuming self-similar characteristics of the pore edge existed in the hydrate media, the following form of expression is used to compute the activity of water (*a*_*w*_) for both the growth sites as:

10 $$\mathrm{ln}({{a}}_{{w}})=\,\mathrm{ln}({{\gamma }}_{{w}}{{x}}_{{w}})-\frac{{{V}}_{{w}}}{{RT}}(\frac{2k}{{r}_{core}^{{\rm{2}}-{{D}}_{{f}}}}\frac{{\sigma }^{\infty }}{(1+\frac{2k\delta }{{r}_{{core}}^{{\rm{2}}-{{D}}_{{f}}}})})$$

where, *γ*_w_ represents the activity coefficient of water (assumed unity)^31^, *x*_*w*_ the composition of water, *V*_*w*_ the molar volume of water, *D*_*f*_ the fractal dimension of the pore edge, *r*_*core*_ the radius of hydrate core, *σ* ^∞^ the interfacial energy between planar interfaces and *δ* the Tolman length. Note that *k* is a linear function of pore radius (*r*_*pore*_) as shown later.

Now, one can simplify the case with considering hydrate formation in the regular pores of the porous materials and on their effective surface. Supposing cylindrical pores and circular pore edge, the following expression^30, 34^ is used to estimate the activity of water (*a*_*w*_) as:

11 $$\mathrm{ln}({{a}}_{{w}})=\,\mathrm{ln}({{\gamma }}_{{w}}{{x}}_{{w}})-\frac{{{V}}_{{w}}}{{RT}}(\frac{2}{{{r}}_{{pore}}}{{\sigma }}_{{H}-{W}})$$

### Predicting real-time formation kinetics

To illustrate the proposed formulation made in the form of a kinetic model and prove its versatility, here the formation of two gas hydrates (CO_2_ and CH_4_) is discussed in three different porous media, namely silica gel, silica sand and hollow silica with a wide range of considerations. For this, the available experimental data sets are used and the model performance is quantified in terms of the absolute average relative deviation (AARD) that is expressed as:

12 $${\rm{AARD}}( \% )=(\frac{100}{{n}_{dp}}\sum _{i=1}^{n}|\frac{W{C}_{e}-W{C}_{p}}{W{C}_{e}}|)$$

where, *n*_*dp*_ is the total number of experimental data points, and *WC*_*e*_ and *WC*_*p*_ are the experimental and model predicted water conversion to hydrates (%), respectively. This *WC* is estimated as:

13 $${\rm{WC}}=100(\frac{{n}_{{\rm{gg}}}{n}_{{\rm{H}}}}{{n}_{{{\rm{H}}}_{{\rm{2}}}{\rm{O}},{\rm{T}}}})$$

The subscript gg, H_2_O and H refer to the guest gas, water and hydrate, respectively. It should be noted that the conditions used in modeling and experiments in references are same.

#### CO_2_ Hydrate

Let us first concentrate on the formation of CO_2_ hydrates in two porous media, namely silica gel and silica sand with three sets of gas compositions as CO_2_-N_2_ (17–83%), CO_2_-H_2_ (40–60%) and CO_2_-H_2_-C_3_H_8_ (38.1–59.4–2.5%).

#### Silica gel

With silica gel and a binary gas mixture of CO_2_-N_2_ (17–83%), the following four versions of the model are proposed to find the best performing case:

*Case I:* Only CO_2_ forms hydrate on the surface and in the irregular pores of the distributed porous particles.

*Case II*: Both CO_2_ and N_2_ form hydrate on the surface and in the irregular pores of the distributed porous particles.

*Case III*: Only CO_2_ forms hydrate on the surface and in the regular pores of the distributed porous particles.

*Case IV*: Both CO_2_ and N_2_ form hydrate on the surface and in the regular pores of the distributed porous particles.

**Figure 2:**
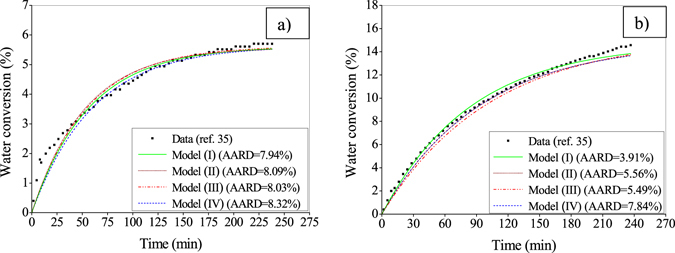
Comparative performance of the four versions of the developed model. For this, the experimental data are adopted from literature^35^ produced at an operating temperature of 272.15 K, and pressure of 8 MPa (Fig. 2a) and 9 MPa (Fig. 2b). Moreover, the runs are performed with a feed gas mixture of CO_2_-N_2_ (17–83%) in the bed of silica gel with 45–75 µm particle size distribution having a pore radius of 30 nm. The percent AARD values are given in the figure against each of the four cases.

Figure 2a and b compare the above four versions of the kinetic model with reference to the experimental data^35^ in terms of water conversion to gas hydrates formed in silica gel at two different operating pressures. In this study, the distributed porous particles are used at their arithmetic mean radius. The model parameters, namely *α*, *β*, Δ*E* and *K*_0_ are optimized using the generalized reduced gradient (GRG) method, and they are reported in Table 1. With this, the best performance is achieved by Case I of the developed model as evident from the AARD value (7.94% in Fig. 2a and 3.91% in Fig. 2b) mainly because of considering irregular pores for hydrate formation and growth. Further, comparing two cases (I-II or III-IV) it becomes obvious that N_2_ does not have any significant role in hydrate formation. In the sequel, thus the best performing version (i.e., Case I) of this model will further be investigated to gain insight into the formation kinetics.

**Table 1:** Optimal model parameters.

	*α*	${{\boldsymbol{K}}}_{{\bf{0}}}^{{\bf{a}}}$	${{\boldsymbol{\Delta }}{\boldsymbol{E}}}_{{\boldsymbol{a}}}^{{\boldsymbol{b}}}{\boldsymbol{\times }}{{\bf{10}}}^{{\boldsymbol{-}}{\bf{3}}}$	*β*
Silica gel/CO_2_-N_2_/8 MPa/272.15 K	0.1092	5.53E-05	10.06	0.5088
Silica gel/CO_2_-N_2_/9 MPa/272.15 K	0.2868	1.01E-05	74.46	0.6032
Silica gel/CO_2_-H_2_/8.5 MPa/274.15 K	0.1161	1.23 E-05	3.91	0.6181
Silica gel/CO_2_-H_2_-C_3_H_8_/4.5 MPa/274.15 K	0.0768	4.92 E-06	5.93	0.3928
Silica gel/CO_2_-H_2_-C_3_H_8_/5.5 MPa/274.15 K	0.0794	1.22 E-05	7.44	0.4966
Silica sand/CO_2_-H_2_-C_3_H_8_/4.5 MPa/274.15 K	0.1240	0.96 E + 02	12.92	0.3942
Silica sand/CO_2_-H_2_-C_3_H_8_/5.5 MPa/274.15 K	0.2409	0.95 E + 02	11.98	0.4766
Silica sand/CO_2_/3.5 MPa/277.2 K	0.4626	0.99 E + 02	32.54	0.4045
Silica sand/CH_4_/8 MPa/277.15 K	0.5567	9.97 E + 02	37.42	0.5485
Hollow silica (1:4)^*^/CH_4_/8 MPa/278.2 K	0.5623	99.89 E + 03	34.95	0.6792
Hollow silica (1:6)^*^/CH_4_/8 MPa/278.2 K	0.5425	10.03 E + 04	33.82	0.5520
Hollow silica (1:8)^*^/CH_4_/8 MPa/278.2 K	0.5443	10.04 E + 04	33.30	0.4819

^*a*^ *K*_*0*_ in mol CO_2_.mol H_2_O^−1^.m^−2^.min^−1^; ^*b*^Δ*E*_*a*_ in J.mol^−1^; ^*^ratio of the mass of hollow silica (g) and the volume of water (ml).

**Figure 3:**
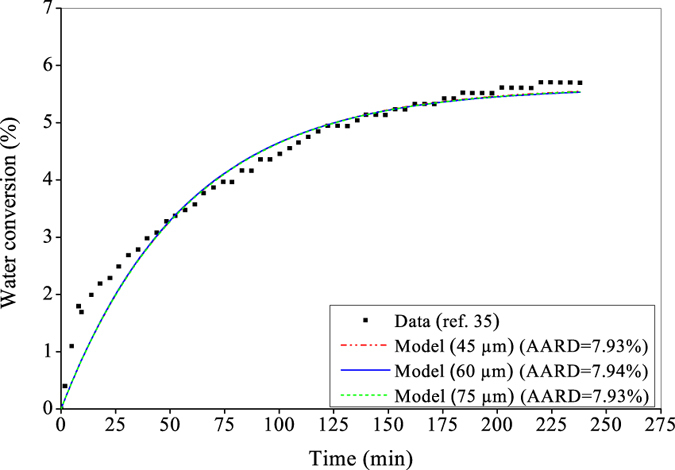
Performance evaluation with CO_2_ hydrate formation in silica gel at a varying particle size. The three sets with a lowest size of 45 µm, average of 60 µm and highest of 75 µm are used for comparison keeping the followings same: pore size at 30 nm, CO_2_-N_2_ composition at 17–83%, pressure at 8 MPa and temperature at 272.15 K.

In the next study, attempt is made to investigate the effect of particle size on water conversion to the hydrate phase. Determining optimal parameter values (Table 2) and then using them, it is investigated in Fig. 3 that the developed kinetic model performs closely, despite a difference existed in particle size. This is achieved by selecting an optimal value for *β* and Δ*E*_*a*_ against each particle size as shown in Table 2. This scope of retuning leads to make the model almost unaffected by the particle size as indicated in terms of AARD values highlighted in Fig. 3 (~7.93% in all three cases).

**Table 2:** Optimal model parameters against size of distributed particles.

Model parameter	Particle size		
	(45 µm)	(60 µm)	(75 µm)
*α*	0.1095	0.1092	0.1094
${K}_{{0}}^{a}$	3.855 E-05	5.53 E-05	8.38 E-05
${\rm{\Delta }}{E}_{a}^{b}$	10000.00	10000.00	10000.00
*β*	0.5289	0.5088	0.4058

^*a*^ *K*_0_ in mol CO_2_.mol H_2_O^−1^.m^−2^.min^−1^; ^*b*^Δ*E*_*a*_ in J.mol^−1^.

**Figure 4:**
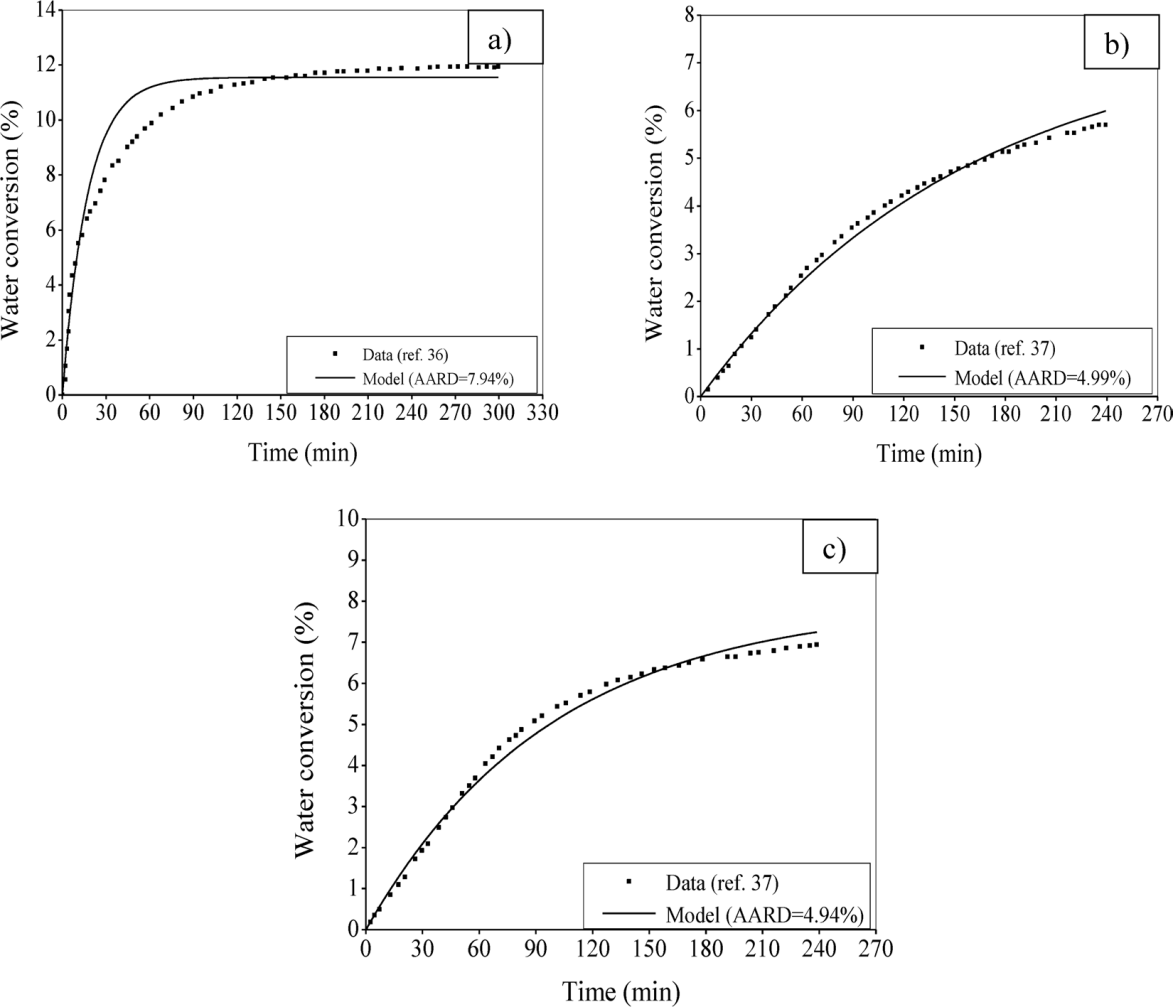
Performance evaluation with CO_2_ hydrate formation in silica gel at different feed gas and operating pressure. The model predicts the experimental data^36, 37^ of water converted to hydrate at 274.15 K. The particles are distributed in the range of 75–100 µm with a pore size of 100 nm. Figure 4a considers the feed gas mixture of CO_2_-H_2_ (40–60%) at operating pressure of 8.5 MPa, Fig. 4b considers the feed gas mixture of CO_2_-H_2_-C_3_H_8_ (38.1–59.4–2.5%) at operating pressure of 4.5 MPa and Fig. 4c is based on the same ternary feed gas mixture at operating pressure of 5.5 MPa.

Further, Fig. 4 evaluates the formulation made in the form of a model for hydrate formation using the experimental data taken from literature^36, 37^. In these experiments, a variation is made in the feed gas along with the operating pressure. For the binary feed consisting of CO_2_ and H_2_ (Fig. 4a) and the ternary feed of CO_2_, H_2_ and C_3_H_8_ (Fig. 4b and c), only CO_2_ forms gas hydrate as indicated before. Along with following the real-time growth trend, the model shows its promising performance in the aspect of the degree of closeness achieved between the predicted and experimental figures. This is reflected through the error calculated in terms of AARD (i.e., 7.94% in Fig. 4a, 4.99% in Fig. 4b and 4.94% in Fig. 4c).

#### Silica sand

**Figure 5:**
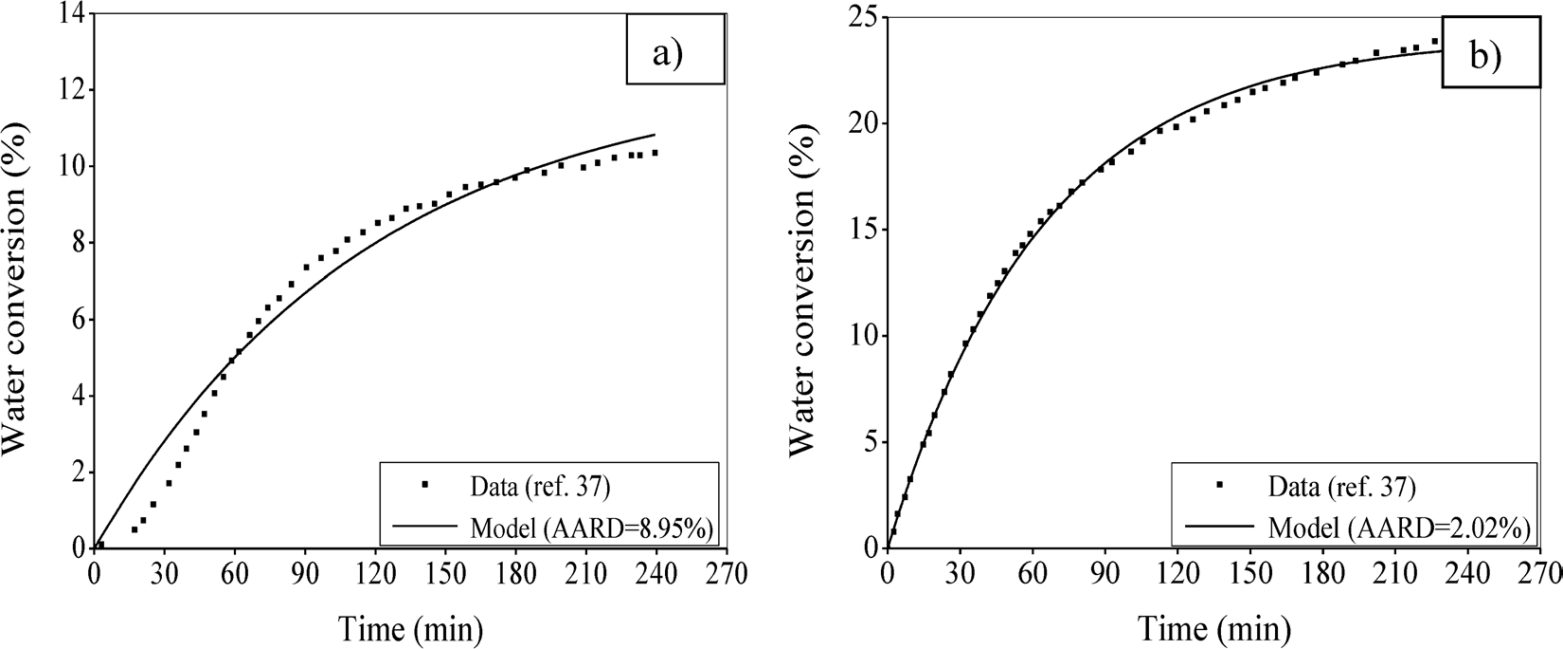
Performance evaluation with CO_2_ hydrate formation in silica sand at different operating pressures. Here, the data sets^37^ are available at 274.15 K in presence of the said porous medium having particles distributed in the range of 150–630 µm with a pore size of 0.9 nm. A feed gas mixture of CO_2_-H_2_-C_3_H_8_ (38.1-59.4-2.5%) is used in the experiment conducted at 4.5 MPa (Fig. 5a) and 5.5 MPa (Fig. 5b).

Now attempt is made to test the model for CO_2_ hydrate formation in a different porous media, namely silica sand. Unlike the silica gel, it has reasonably small pores. Here, two sets of results are produced in Fig. 5a and b, which are different in terms of their operating pressures. Finding the optimal parameter sets (Table 1) and using them, it is evident that the proposed formulation is capable enough in predicting the real-time formation behavior. In this regard, one can see the AARD values provided in the figure itself.

It is observed from the experimental investigation^37^ that to a certain extent, the initial rate of water conversion increases with the increase of pressure. The subsequent parts of the conversion rate profiles follow the similar trend but with a larger magnitude at a higher pressure. Now, one can closely observe that the real-time water conversion at 4.5 MPa (Fig. 5a) is relatively slow at the beginning and then it picks up speed. This typical behavior (sigmoidal shape) can be approximated by the response of a first-order system with a time lag. On the other hand, the water conversion at 5.5 MPa (Fig. 5b) leads to the response of a first-order system only (negligible time lag). Now, the governing equation in the proposed model [i.e., Equation (7)] is quite similar to the model of a first-order system (with no time lag) and thus, the model predicts the conversion rate at 5.5 MPa better than that at 4.5 MPa. However, this difference in initial conversion rate between 4.5 and 5.5 MPa does not exist for silica gel as shown in Fig. 4b and c before, and thus, the AARD values are quite close between them.

**Figure 6:**
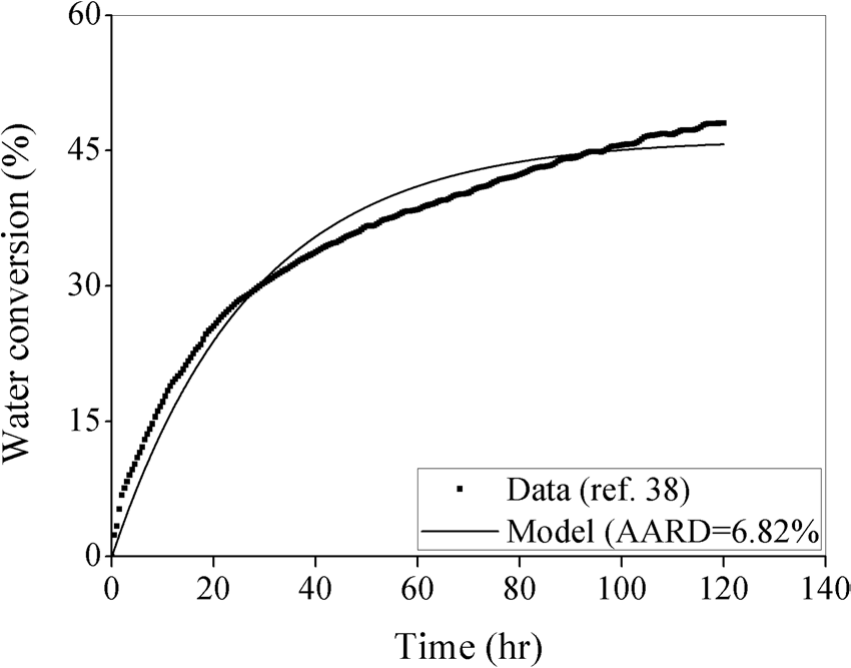
Performance evaluation with CO_2_ hydrate formation in silica sand. For this study, the data sets^38^ are available at 277.2 K in presence of the said porous medium having particles distributed in the range of 100–500 µm with a pore size of 0.9 nm. A pure CO_2_ (99.9%) is used in the experiment conducted at 3.5 MPa.

It is fairly true that the natural hydrate formation process continues for a long period of time. Keeping this issue in mind, the model predictability is further tested in Fig. 6 for continuously about 120 hour (5 days). In case of pure CO_2_ gas (99.9%), the experimental data are taken from literature^38^. It is evident that the proposed model shows a good prediction with a reasonably low AARD of 6.82%.

#### CH_4_ Hydrate

To evaluate the developed formulation and its versatility, further the formation and growth of CH_4_ hydrate are considered in presence of two porous media, namely silica sand and hollow silica. As stated earlier, the same experimental conditions are used in the model simulation.

#### Silica sand

**Figure 7:**
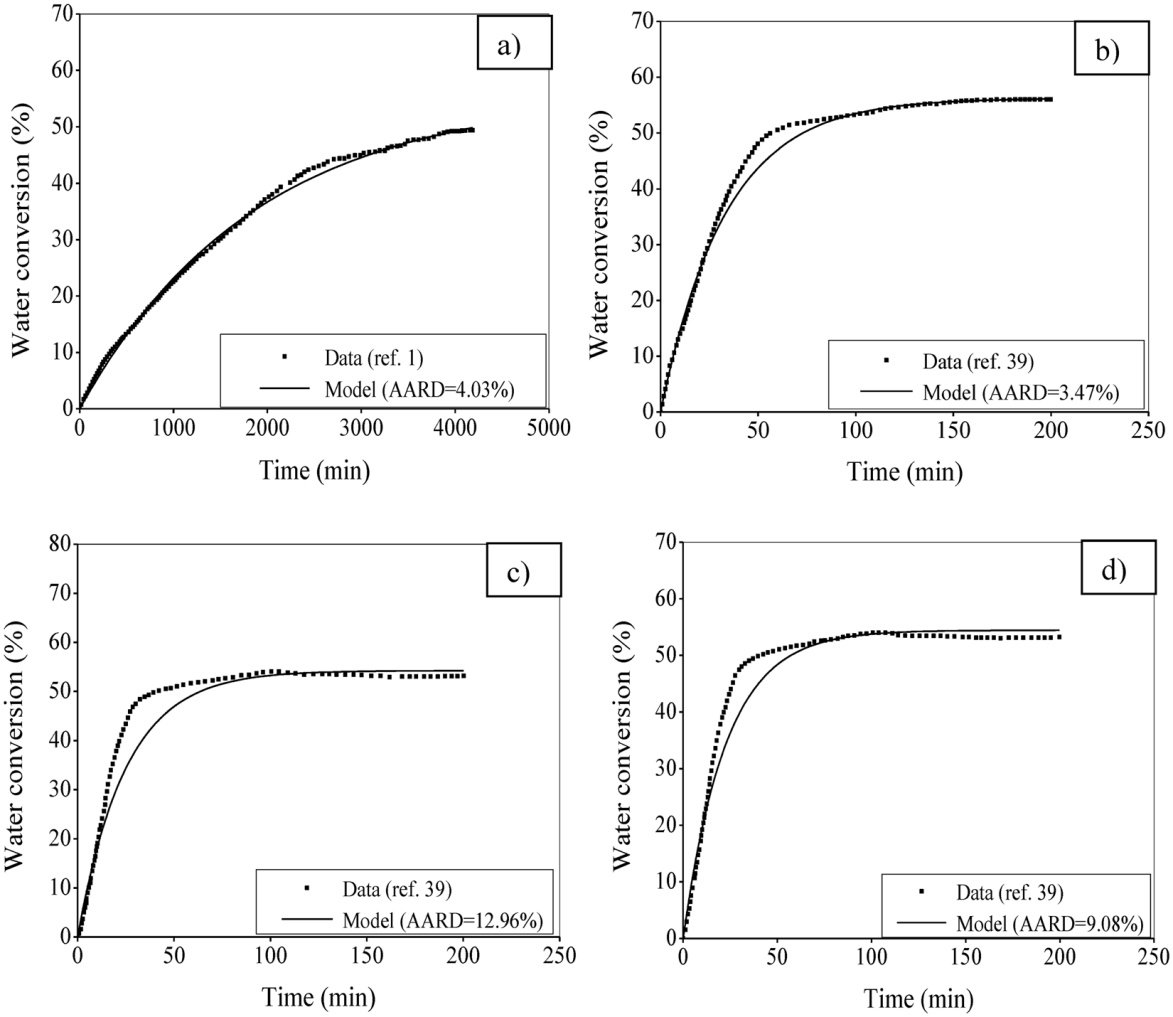
Performance evaluation with CH_4_ hydrate formation in silica sand (Fig. 7a) and hollow silica (Fig. 7b–d). Experimental data are available in literature^1, 48^ for a pure CH_4_ gas fed to perform the runs at 8 MPa pressure.

Figure 7a depicts the performance of the kinetic model with reference to the experimental data^1^ in the aspect of water conversion to CH_4_ hydrate in silica sand. The pure CH_4_ gas is used for the hydrate formation and subsequent growth. This study is performed at 8 MPa and 277.15 K with the average particle size chosen for the distributed range of 560–1300 µm. The pore size is considered as 0.9 nm. Using the identified model parameters (Table 1), the model shows an excellent agreement with the data with an AARD of about 4%. This is achieved by addressing a couple of practical issues in the model formulation as stated before. It should be noted that this test is conducted for a long period of time (about 4200 min ( = 70 hour)) typically involved in the natural hydrate formation process.

#### Hollow silica

To predict the real-time kinetic profile of CH_4_ hydrate growth, the formulation made in this study is used for the hollow silica distributed in the range of 30–70 µm. The system operates at 8 MPa and 278.2 K. Taking average particle size, the ratio of the mass of hollow silica and the volume of water is considered as 1:4, 1:6 and 1:8 in Fig. 7b,c and d, respectively. From the results, one can see the promising performance of the model, indicating that the formulation has well taken the issues that have practical relevance.

## Discussion

Here, a physical model is developed to understand the hydrate formation phenomena. This model is novel in that it considers the clathrate hydrate formation in both the interstitial pore space between porous materials and inside the nanometer-sized pores of those materials. By considering chemical potential as a driving force for growth, the combined effect of temperature, pressure and composition is taken into account. More importantly, the proposed formulation addresses a couple of practical issues concerning irregularity in the pores of distributed particles, surface tension effect in the pores, among others. Excellent agreement is achieved between the model prediction and experimental data for several porous media, and this is also reflected through the AARD values. Thus, it can be concluded that the proposed formulation is rigorous and versatile enough to represent a generalized model in predicting the formation kinetics of clathrate hydrates. This model can further be improved by renewing the surface area of the water in contact with the hydrate gas with time, and considering pore size distribution in the non-spherical porous particles.

## Methods

### Estimating ${{\boldsymbol{\mu }}}_{{\boldsymbol{w}}}^{{\boldsymbol{A}}}$

To use Equation (9) that models ${\mu }_{{\rm{w}}}^{{\rm{A}}}$, one needs to estimate $\Delta {h}_{w}^{{\rm{A}}}$, for which, the following form is recommended,

14 $${\rm{\Delta }}{h}_{{\rm{w}}}^{{\rm{A}}}(T)={\rm{\Delta }}{h}_{{\rm{w}}}^{{\rm{0}}}({T}_{0})+{\int }_{{T}_{0}}^{T}{\rm{\Delta }}{C}_{{\rm{pw}}}^{{\rm{L}}}{\rm{d}}T$$

in which, ${\rm{\Delta }}{h}_{{\rm{w}}}^{{\rm{0}}}({T}_{0})$ is estimated at reference temperature and absolute zero pressure, and its value is adopted – 4709.5 J.mol^−1^ (ref. 31). Here, ${\rm{\Delta }}{C}_{{\rm{pw}}}^{{\rm{L}}}$ is the heat capacity difference between empty hydrate lattice and liquid water^31^.

To model the activity of water (*a*_*w*_) in a porous medium, the following form^30^ is used:

15 $$\mathrm{ln}({a}_{w})=\,\mathrm{ln}({{\gamma }}_{{w}}{{x}}_{{w}})+\frac{{V}_{w}}{RT}(-{\rm{\Delta }}P)$$

where, Δ*P* denotes the difference in pressure between the aqueous and hydrate phase. *x*_*w*_ is determined based on the guest gas present in aqueous solution (*x*_gg_) as:

16 $${{x}}_{{w}}=1-{x}_{{\rm{gg}}}$$

At equilibrium,

17 $${\mu }_{{\rm{gg}}}^{{\rm{A}}}={\mu }_{{\rm{gg}}}^{{\rm{V}}}$$

Here, ${\mu }_{{\rm{gg}}}^{{\rm{A}}}$ and ${\mu }_{{\rm{gg}}}^{{\rm{V}}}$ are the chemical potential of guest gas in the aqueous and vapour phase, respectively. Now one can calculate *x*_gg_ by estimating the molality of the respective guest gas (*m*_gg_) from the following equation:

18 $$\mathrm{ln}(\frac{{y}_{{\rm{gg}}}P}{{m}_{{\rm{gg}}}})=(\frac{{\mu }_{{\rm{gg}}}^{A(0)}-{\mu }_{{\rm{gg}}}^{V(0)}}{RT})-\,\mathrm{ln}\,{\varphi }_{{\rm{gg}}}+\,\mathrm{ln}\,{\gamma }_{{\rm{gg}}}$$

with

19 $${x}_{{\rm{gg}}}=\frac{{m}_{{\rm{gg}}}}{{m}_{{\rm{gg}}}+{m}_{{\rm{w}}}}$$

where, *m*_w_ is the number of moles of water per kg of water (55.56 mol/kg)^31^, *y*_gg_ the mole fraction of guest gas in vapour phase, ${\mu }_{{\rm{gg}}}^{A(0)}$ and ${\mu }_{{\rm{gg}}}^{V(0)}$ the standard chemical potential of a guest gas in the aqueous and vapour phase, respectively, *ϕ*_*gg*_ the fugacity coefficient of guest gas that is estimated here by using the Soave-Redlich-Kwong equation of state^39^, and *γ*_gg_ the activity coefficient of guest gas (assumed as one)^40^. Note that the ${\mu }_{{\rm{gg}}}^{V(0)}$ is adopted as zero^31^.

The ${\mu }_{{\rm{gg}}}^{A(0)}$ is a function of operating temperature and pressure, and it is calculated by the following equation for methane hydrate^31^:

20 $$\frac{{\mu }_{{{\rm{CH}}}_{{\rm{4}}}}^{{\rm{A}}(0)}}{RT}={C}_{1}+{C}_{2}T+{C}_{3}/T+{C}_{4}{T}^{2}+{C}_{5}/{T}^{2}+{C}_{6}P+{C}_{7}PT+{C}_{8}P/T+{C}_{9}P/{T}^{2}+{C}_{10}{P}^{2}T$$

in which, *C*_1_ to *C*_10_ are the coefficients. Similar form of equations is also reported for other gas hydrates^40, 41^.

As far as Δ*P* is concerned, it is proposed to consider the growth of hydrate in irregular nanometer-sized pores. For this, the following form^33^ is used:

21 $$\Delta P=\frac{L}{S}{{\rm{\sigma }}}_{{\rm{H}}-{\rm{A}}}\,\cos \,{\rm{\theta }}$$

in which, σ_H−A_ is the surface tension of water between aqueous and hydrate phase, and *θ* the contact angle between water and porous media, which is zero. Here, *L* and *S* are the perimeter and area of the pore edge for the irregular capillaries, respectively. According to the fractal theory^42^, *L* can be calculated using the fractal dimension of the pore edge (*D*_*f*_) and pore radius (*r*_*pore*_) as:

22 $$L=2\pi k{r}_{pore}^{{D}_{f}}$$

where, *k* is a linear function of *r*_*pore*_,

23 $$k=a{r}_{pore}+b$$

in which, *a* and *b* are the coefficients that can be estimated from the experimental data^33^. Here, it is supposed^33^ that the area of hydrate core, *S* is same as that of circular hydrate core. Similarly, the perimeter of hydrate core (*l*) (Fig. 1b) can be expressed in terms of *r*_*core*_ as:

24 $$l=2\pi k{r}_{core}^{{D}_{f}}$$

Further simplifying and rearranging, Δ*P* can be expressed as:

25 $${\rm{\Delta }}P=\frac{2k}{{r}_{core}^{2-{D}_{f}}}{\sigma }_{{\rm{H}}-{\rm{A}}}$$

in which, σ_H−A_ is represented as follows^43^:

26 $${\sigma }_{{\rm{H}}-{\rm{A}}}=\frac{{\sigma }^{\infty }}{1+\kappa \delta }$$

where, *σ* ^∞^ is adopted as 0.0267 J.m^−2^ (ref. 44). The thickness of an interfacial region between solid (ice) and aqueous (water) phase (*δ*) is commonly referred as Tolman length, which is equal to 0.4186 nm^45^. In addition, the solid-liquid interfacial curvature (*κ*) is considered as a function of *r*_*core*_ and *D*_*f*_, and it is given as^33^:

27 $${\rm{\kappa }}=\frac{2{\rm{k}}}{{{\rm{r}}}_{{\rm{core}}}^{{\rm{2}}-{{\rm{D}}}_{{\rm{f}}}}}$$

Substituting Equations (25–27) in (15), one obtains the model of *a*_*w*_ represented in Equation (10).

To simplify the case with hydrate formation in the regular pores and on the effective surface of the porous particles, it assumes cylindrical pores and circular pore edge, for which^30^,

28 $$\Delta P=\frac{2}{{r}_{pore}}{\sigma }_{{\rm{H}}-{\rm{A}}}\,\cos \,\theta $$

Now, substituting Equation (28) in (15), one can obtain the corresponding model Equation (11) for *a*_*w*_.

### Estimating ${{\boldsymbol{\mu }}}_{{\boldsymbol{w}}}^{{\boldsymbol{H}}}$

For Equation (8) that models ${\mu }_{{\rm{w}}}^{{\rm{H}}}$, the Langmuir type expression is used to obtain *θ*_*ij*_ as^30^:

29 $${\theta }_{ij}=(\frac{{C}_{ij}\,{f}_{j}}{1+\sum _{j=1}^{{N}_{c}}{C}_{ij}\,{f}_{j}})$$

in which, *C*_*ij*_ represents the Langmuir constant of gas component *j* in an *i* type cavity. The fugacity of gas component *j* in the hydrate phase (*f*_*j*_) is estimated from the Soave-Redlich-Kwong (SRK) equation of state. This *f*_*j*_ is assumed same with the fugacity of component *j* in the gas phase^46^. Now, *C*_*ij*_ is computed from^30^:

30 $${C}_{ij}=\frac{4{\pi }}{KT}{\int }_{0}^{R}\exp (\frac{-\omega (r)}{KT}){r}^{2}{dr}$$

Here, *K* denotes the Boltzmann’s constant, *R* the cell radius of hydrate and *ω*(*r*) the spherically symmetric cell potential, which is obtained from^47^:

31 $$\omega (r)=4\varepsilon [{(\frac{\sigma -2a}{r-2a})}^{12}-{(\frac{\sigma -2a}{r-2a})}^{6}]$$

where, the constants^47^, *ε*, *σ* and *a* denote the maximum attractive potential, the cores distance at zero potential and the radius of the spherical core, respectively.

### Estimating particle surface area for hydrate growth

Along with the pores, as stated, the surface of the porous particle is also involved in hydrate formation and growth. In this light, the concept of effective surface area (*A*_*e*_) is introduced in the proposed model. Now the total surface area of the porous material (*A*) is estimated by multiplying the individual particle surface area (*A*_*pi*_) with the total number of particles (*n*_*tp*_) present in the bed as:

32 $$A={n}_{tp}{A}_{pi}$$

Usually, the size of the porous material is known in terms of its diameter (*d*_*p*_) and hence, it is easy to find the *A*_*pi*_ assuming spherical particle from:

33 $${A}_{pi}=\pi \,{d}_{{\rm{p}}}^{{\rm{2}}}$$

On the other hand, *n*_*tp*_ is obtained from:

34 $${n}_{tp}={V}_{tp}/{V}_{pi}$$

where, *V*_*tp*_ denotes the total volume of the porous media and *V*_*pi*_ the volume of a single particle. The *V*_*tp*_ is calculated by subtracting the volume of water required to fully saturate the fixed bed (*V*_*ws*_) from the total volume of the bed (*V*_*b*_). Knowing *V*_*ws*_, one needs to determine *V*_*b*_ from:

35 $${V}_{b}=\pi {d}_{b}^{{\rm{2}}}{h}_{b}$$

Here, *d*_*b*_ and *h*_*b*_ are the diameter and height of the fixed bed of a porous medium.

### Data availability

The data sets that support the findings of this work are available from the corresponding author upon request.
